# Development of multifactor index for assessing quality of life of a tribal population of India: multilevel analysis approach

**DOI:** 10.1186/s12889-021-10338-2

**Published:** 2021-02-18

**Authors:** M. Bagavandas

**Affiliations:** Centre for Statistics, SRM Institute of Science and Technology, Kattankulathur, Tamil Nadu 603203 India

**Keywords:** Quality of life, Multi-factor index, Multilevel analysis, Factor analysis, Eigen value, Nested data, Domains, Households and villages

## Abstract

**Background:**

The main objective of this study is to develop a multilevel multi-factor index to assess the quality of life of the Malayali tribal population of India at the household and village levels based on nine domains, namely, Demography, Economy, Health, Human Development, Infrastructure Development, Work Participation, Recreation, Social Capital and Self Perception. An attempt is made to classify the individuals as well as villages by the overall scores of a multi-factor -index within a community which will help policymakers to develop concrete policy recommendations for the improvement of the quality of life of this tribal group.

**Method:**

Multilevel factor analysis is utilized to determine uncorrelated meaningful factors and their respective weights using Mplus software from the nested dataset consists of values of nine domains of 1096 individuals collected from 19 villages. A multilevel multi-factor index is constructed using the weights of these factors. The qualities of the lives of different households and different villages are assessed using the scores of this index.

**Results:**

Three different factors are identified at household as well as village levels. The quality of life at Households and Village levels are classified as poor, low, moderate, good, and excellent based on five quintiles of the scores of the multi-factor index, and the contribution of each domain in this classification is ascertained.

**Discussion:**

This study finds that at household as well as at village levels, the quality of life of the individuals of this tribal population increases with an increase in education, income, and occupation status which make them lead a healthy life and also make them to find time and money to spend on recreation. Infrastructure is not important at the household level but not so at the village level.

**Conclusion:**

The main purpose of developing this kind of multi-factor index at different levels is to provide a tool for tribal development based on realistic data that can be used to monitor the key factors that encompass the social, health, environmental, and economic dimensions of quality of lives at the household and community levels of these tribal people.

**Supplementary Information:**

The online version contains supplementary material available at 10.1186/s12889-021-10338-2.

## Background

Quality of life describes the well-being of a human’s life. The concept of quality of life varies from an individual’s feelings of wellbeing to mental, social, occupational, spiritual, marital, and physical functioning [1]. Dissart and Deller (2000) argued that an individual’s quality of life depends on the objective facts of life as well as the subjective perceptions of these factors [2]. Over the years,the concept of quality of life has given image makeover from health-related factors to non-health-related issues like the standard of living, subjective wellbeing, happiness, human development, gender development, education, recreation, and leisure [3, 4]. The main idea of assessing the quality of life is to create an opportunity for individuals to live longer with healthy, creative, and satisfying lives in a good environment. Measuring the quality of life of society will help the development authorities to identify the problematic areas and provide effective management suggestions for the improvement in the well-being of individuals of that society [5].

The number of research papers on quality of life has been increasing steadily. Literature survey says that the concept of quality of life depends on the area of research and the type of problem to be discussed [6, 7]. Kane (2001) identified that factors like the sense of safety, security and order, physical comfort, enjoyment, meaningful activity, relationships, functional competence, dignity, privacy, autonomy, individuality, and spiritual well-being define the quality of life [8]. Noronha and Nairy (2005) have defined quality of life as the satisfaction of needs, feeling of well- being, and working conditions [9]. Phillips (2006) defined quality of life as the autonomy to choose to enjoy life, to flourish, and to participate as citizens in a society with high levels of civic integration, social connectivity, trust, and other integrative norms. According to the International Society for quality-of-life studies (2007), quality of life is the degree to which a person’s life is desirable, often with an emphasis on an internal component such as environment and income [10, 11]. Quality of life is defined as an individual’s perception of their position in life in the context of the culture and value systems in which they live and concerning their goals, expectations, standards, and concerns [12].

The quality-of-life index is a composite criterion that consists of certain social, physical, economic, and psychological factors to measure the well-being of groups of individuals or communities and is considered as a tool in policy analysis and public health administration [13]. Initially, objective factors based on indices like GDP, Human Development Index, and Wealth Index have been developed and of late more subjective factors based on indices such as subjective wellbeing index, emotional wellbeing index, happiness index, etc. are being increasingly included in the measurement of quality of life along with objective indices [14]. The quality of life index encompasses different objective and subjective dimensions and if these dimensions are aggregated properly then an overall value of quality of life of individuals or communities can be derived.

Nowadays enough literature is available on the choice of indicators to be used to measure the quality of life. These indicators represent individual, interpersonal, and contextual aspects of quality of life. Discussions on developing indices for assessing the quality of life have led to numerous important initiatives undertaken by different scholars worldwide for decades [15]. These exercises led to a growing consensus on the need for a comprehensive wide-ranging data-driven approach for developing indices that encompass all aspects of life to define and measure the quality of life of different societies and finally applicable for both analytical and policy-making purposes [16]. These indices are periodically used in measuring the progress of communities and their well-being and in developing methodologies for sustaining the quality of life for the future [17].

Saharnaz Nedjat’s (2011) study on the Iran population indicates that factors like age, sex, and education, and employment status play major roles in determining the quality of life [18]. A systematic review study on the quality of life of the general population reveals that large proportions of the population are satisfied with their lives in ‘general’ terms and the ratings of quality of life are frequently highest in family and lowest in finances; personal characteristics and objective circumstances are not a major influence on subjective evaluations of the quality of life in general [19]. Victoria Cramer et al. (2004) find that good somatic health, living with a stable partner in a less densely populated area, having a good education and good income determine the global quality of life [20]. Population density, psychological, relational, and environmental factors influence the quality of life of citizens of Northern Italy people [21]. Fiona Y Wong et al., (2018) by determining the association between quality of life and neighborhood environment satisfaction of residents of Hong Kong city concluded that this type of study provides policymakers and health administrators with evidence-based information on how physical and built environment can influence the quality of life of the residents which facilitates the environment interventions and policy recommendations [22].

This study attempts to assess the quality of life of the Malayali tribal population of Tamil Nadu State which is situated in the southern part of India. This group is the largest tribal population of this state. Mostly they live in hilly regions and they live in the condition of isolation from their socio-cultural system [23].

Studies on the assessment of the quality of life of Indian tribal populations are limited but increasing steadily. Quality of life of the tribal population of Kerala State, India was assessed using deprivation index and inferred that the level of deprivation in terms of housing, basic facilities, and economic status is very high compared to the general population of the same state [24]. Jana and Prasanta Kumar Ghosh (2015) [25] have analyzed various socio-economic indicators to assess the quality of life of the tribal population of Mayurbhanj District of Odisha State of India and inferred that the quality of life of this population has remarkably improved over the years but there exist economic disparities in terms of gender and caste. Secondary data analysis of different elderly tribal populations assessed the security index based on both financial and social security status and identified that security becomes poorer as age increases, moderate index values decrease as age increases, and better security is also found among the oldest old [26].

The main objective of this study is to develop a multilevel multi-factor index to assess the quality of life of the Malayali tribal population at the individual and village levels to determine the roles of different indices defined at multiple levels in shaping the quality of life of this tribal population. It will attempt to classify the individuals and villages based on this overall performance of the multi-factor index within a community which will help policymakers to develop concrete policy recommendations for the improvement of the quality of life of this tribal group. Demography, Health, Economic Condition, Infrastructure Development, Recreation, Work Participation, Human Development (Wealth Status and Literacy Rate), Social Capital, and Perceived Quality of life are the nine domains considered for this study. This tool can be expanded and tested on various other tribal communities by making adjustments to suit their locally available conditions. The different domains for this study were selected based on the article entitled “A Survey of Composite Indices Measuring Country Performance: 2006 Update” written by Romina Bandura and Carlos Martin (2006). A study was conducted for the first time in India to characterize the socio-economic distribution of health in India as measured by life expectancy at birth and in so doing quantifies health inequalities occurring across the lives of the Indian population [27]. The Kuppusamy scale was used to assess the socio-economic status of school-going children in North Bengaluru, India to assess its influence along with environmental factors on their oral health [28].

## Methods

Table 1 provides the details regarding the domains,indicators, and variables used for this quality assessment process.

**Table 1:** The details of Domains, Indicators, and Variables considered for this study

Domains	Indicators with their variables
Demography	Family Size, Type of Family, Mortality Rate and Sex Ratio
Health	Morbidity Rate, Ante Natal Care, Delivery Care, and Post Natal Care, Breast Feeding Pattern and Vaccination Coverage
Economic Status	Education, Occupation, and Income of Individuals
Infrastructure Development	Availability of the following facilities in the village- Sanitation, Electricity, Communication, and Transport
Work Participation	Employment and Self-employment Rates- Working Population at the specific age group
Social Capital	Membership- Membership in various Social Groups; Participation- Their Roles and Responsibilities in Social Groups; Reciprocity- Mutual Exchange of Commercial or other Privileges
Recreation	Participation in Recreation and Leisure Activities
Human Development-Wealth Status	Ownership of the house; 2. The number of rooms; 3. Type of house; 4. Fuel used for cooking; 5. Source of lightning; 6. Availability of kitchen; 7. Radio; 8. Television (colour); 9. Landline; 10. Mobile phone; 11. Ceiling Fan; 12. Table fan; 13. Chair; 14. Cot/Bed; 15. Mattress; 16. Sewing machine; 17. Pressure Cooker; 18. Grinder; 19. Mixie; 20. DVD/VCD Player; 21. DTH; 22. Table; 23. Wall Clock; 24. Wardrobe; 25. Bicycle; 26. Water Pump; 27. Motorcycle or Scooter; 28. Lands (Wet & Dry); 29. Cows/Buffalos/Bulls; 30. Goats; 31. Sheep; 32. Chickens/Ducks; 33. Pigs; 34. Source of water; 35. Toilet facilities; 36. Drainage Type;
Human Development Literacy Rate	Can Read and Write with specific age Group in each household
Self-Perception Rate	Self-Perception about quality of life

### Multi-factor index

The most common and simplest way to construct a multi-factor index is to take a weighted average of two or more single factor indices and such indices are constructed by combining several such indicators for evidence-based decision making [29]. Quality of life can be considered as components of different domains and if they are aggregated scientifically then an overall value or score for quality of life can be derived [30]. Researchers have been developing different multi-factor indices based on the choice of indicators that suits to measure various aspects of quality of life in such a way that each indicator is supposed to reflect the magnitude of a specific domain of quality of life [30]. Nowadays, many international institutions are trying to define the quality of life as a component of various aspects such as income, jobs, cost of living, education, environment, and safety to assess individual and social well-being [31]. A multi-factor/composite indicator generally provides better results than a single indicator for a specified subject [32]. Saisana et al. (2005) have opined that a multi-factor index can be used to measure multidimensional issues and facilitates the ranking of communities or countries [33]. The well-known composite index is the Human Development Index developed by the United Nations Development Program which combines education, health, and income [34]. The hospital performance index comprises of bed occupancy rate, bed turnover, and the average length of stay [35]. Other examples are the Physical quality of life index [36], Monetary condition index [37]. This study proposes to use a multilevel factor analysis technique to construct a multi-factor index based on the above said nine domains.

### Construction of multi-factor index

When the data are collected from different individuals within a group, then that data is said to be nested within that group and data are available at different levels. In our case household (level1) data are nested within the village (level 2) they live and these observations need not be independent because all the households may use the same facilities available in that village. In this nested data, the total variance is split into variances within villages and between villages. Moreover, among the nine indicators of this study, some are individually based and some are village-based. For example, literacy rate and wealth indicators are individuals based whereas Infrastructure is a village-based indicator. In such a situation, it is not advisable to use commonly used analytical methods that might produce inaccurate readings of statistical significance. In such a case multilevel analysis is suitable to analyze this kind of structured dataset and it can also provide scores to represent both within individual differences and between-individual differences [38].

Factor analysis is the commonly used multivariate technique to construct a multi-Factor index. The major disadvantage of this method is that it extracts components from the total correlation matrix ignoring the dependency factor present in the data. In such a case the standard errors of parameter estimates and the model fit statistics may be misleading and the component structure may not be correct because it is contaminated by two sources of variance [39]. In such a situation multilevel factor analysis is recommended because it not only produces unbiased estimates of the parameters but also allows discussing village characteristics in the factor structure of individual outcomes [40–44]. This analysis breaks down the total variance-covariance matrix of variables measured at individual level into within village (level 1) and at village level between village matrices and provides factor structure at each level [43–45].

Multilevel factor analysis (MFLA) develops one of the two types of latent constructs based on these indicators such as individual-level constructs that capture the individual quality of life and [2] aggregated scores that capture the quality of life at the village level. This analysis provides two different latent factor structures at two levels, which help us to understand the variation in structure, and meaning that exists between individuals within a village, as well as between villages, rather than assuming that the factor structure is the same at both levels [44].

This paper proposes to identify the domains which play a vital role in assessing the quality of life at the individual level and the village level. This paper proposes to use MLFA [46–48] to assess the quality of life-based on nine domains described earlier. Just like simple factor analysis, MLFA also tries to capture the shared variance among an observed set of variables in terms of a potentially smaller number of unobserved constructs or latent factors [49, 50]. However, MLFA splits the total sample variance-covariance matrix into within-group (i.e., individual level, within a village) and between-group (i.e., village level) matrices and also identifies distinct latent factor structures at each of these levels [45]. This analysis helps the researchers to understand the variations in structures between individuals within a village, as well as between villages.

### Tool Development

After identifying the main domains for assessing the quality of life, the various indicators used to define each domain, and the variables used to define each indicator were identified. Two different questionnaires, namely, the household questionnaire and village questionnaire were developed to quantify the variables and then the indicators of this study to assess the quality of life of this tribal population at the individual level and then at the village level. Experts from different fields such as public health, sociology, anthropology, and statistics have reviewed the questionnaires based on various aspects such as appropriateness, relevance, representativeness, difficulty, and comprehensibility of the items. They were further refined based on the comments of these experts. The tool was then translated to Tamil, the local language, and back-translated to English to check the validity of the translation. This tool was initially pilot-tested on a small sample of 50 households of another tribal population. The time is taken to administer the tools and the ease of administration were assessed. The tool was further refined based on the findings of the pilot test.

### Sample size

The sample size was calculated using Epi Info software version 3.2.5 assuming the following parameters: 1) Tribal population in Tamil Nadu 651,321 [23], 2) Literacy rate among tribal – 41.5%, 3) Absolute precision of estimate 5% and 4) Confidence levels – 95%.

The sample size was 2151 adults. An oversampling of 5% was added to account for absence or non-response. The overall sample size was 2259 adult tribal individuals. Assuming on an average 2 adult members per household this sample size can be achieved by visiting 1130 households.

### Sampling methodology

A systematic, Multi-stage sampling design was adopted. The selection process of the sample was as follows:

i. In the first stage, to capture the uniqueness of tribes; villages, where more than 80% of the Malayali tribe live, were selected based on the information available with the 2011 census. At the second stage, nineteen villages were selectedusing probability proportion to size.
ii. The number of respondents per village was determined based on the population available in the village.
iii. At the third stage, the Circular Systematic Random Sampling (CSRS) was adopted to achieve the sample size in each village. The survey was started at the north-west corner of each village, at first one household was selected at random, and then the other households were selected subsequently using the CSRS method. No replacement was made if the selected household was locked or empty during data collection.

### Data collection

A team of investigators was recruited and given rigorous one-week training on House Listing Activities, Sampling Methodology, and Questionnaires, Types of Respondents, Investigation Ethics, Interview Techniques, and Data Collection Instruments. The data were collected on palmtop computers using Epi Info version 3.2.5 datasheets. Two teams were formed and each team comprised of one supervisor and four investigators. It was decided to interview one individual either husband or wife in each household. All eight investigators were involved in data collection and the supervisors were responsible for online and offline data quality checking. The data cleaning process reduced the sample size from 1130 to 1096 households.

## Results

### Development of multilevel multifactor index

The Mplus software [51] developed by Muthen is used for developing a multilevel multifactor index. The specialty of this powerful package is that it estimates statistical models for observed as well as unobserved (latent) variables separately in four stages [42, 43]. At first, assuming all observations are independent, ordinary factor analysis is conducted on a total correlation matrix of nine domains to get a rough idea about the underlying factor structure. The Intra correlation coefficient (ICC) for each domain is obtained in the second step. The main idea of calculating ICC is that it will help us to determine whether our data need multilevel factor analysis. The third and fourth steps, respectively, involve getting estimates for within-correlation and between-correlation matrices and conduct factor analysis for each matrix separately.

Exploratory factor analysis was made on a total correlation matrix (Table 2) using SPSS (Trial Version) software and the result of this analysis indicates the existence of four factors. This analysis is technically incorrect because this analysis assumes that the between-correlation matrix is a zero matrix. That is, the analysis of the total correlation matrix assumes that there exist no reliable between-individual differences present in the data. To explore the extent to which this is true or false, the intra correlation coefficients are computed for each of the nine indices. The intra correlation coefficients are given in Table 3.

**Table 2:** Total, within and between correlation matrices

	Self- Perception	1								
**Total correlation matrix**	Recreation	0.06	1							
	Social Capital	0.11	0.198	1						
	Infrastructure	−0.081	−0.125	− 0.165	1					
	Human Development	0.037	0.242	0.164	−0.313	1				
	Demography	0.062	0.023	0.207	−0.381	0.245	1			
	Health	0.413	0.018	0.064	−0.02	−0.051	0.061	1		
	Economic	−0.015	0.086	0.011	−0.24	0.386	0.159	0.049	1	
	Work Participation	0.004	0.03	0.051	−0.368	0.314	0.27	−0.022	0.449	1
**Pooled within-sample correlation matrix**	Self- Perception	1								
	Recreation	0.065	1							
	Social Capital	0.098	0.221	1						
	Infrastructure	−0.04	−0.156	−0.14	1					
	Human Development	0.032	0.218	0.169	−0.345	1				
	Demography	0.062	0.047	0.198	−0.385	0.274	1			
	Health	0.418	0.005	0.072	−0.016	−0.062	0.065	1		
	Economic	−0.013	0.073	0.022	−0.263	0.384	0.172	0.039	1	
	Work Participation	−0.003	0.032	0.038	−0.366	0.318	0.249	−0.023	0.46	1
	Self-Perception	1								
	Recreation	0.065	1							
	Social Capital	0.098	0.221	1						
	Infrastructure	−0.04	−0.156	−0.14	1					
	Human Development	0.032	0.218	0.169	−0.345	1				
	Demography	0.062	0.047	0.198	−0.385	0.274	1			
	Health	0.418	0.005	0.072	−0.016	−0.062	0.065	1		
	Economic	−0.013	0.073	0.022	−0.263	0.384	0.172	0.039	1	
	Work Participation	−0.003	0.032	0.038	−0.366	0.318	0.249	−0.023	0.46	1
**Estimated between-sample correlation matrix**	Self-Perception	1								
	Recreation	−0.248	1							
	Social Capital	0.691	−0.599	1						
	Infrastructure	−0.904	0.381	−0.782	1					
	Human Development	0.093	0.586	−0.026	0.036	1				
	Demography	0.243	−0.392	0.712	−0.585	−0.07	1			
	Health	0.06	0.676	−0.422	−0.084	0.418	−0.068	1		
	Economic	−0.149	0.651	−0.599	0.209	0.62	−0.357	0.834	1	
	Work Participation	0.355	−0.166	0.716	−0.634	0.218	0.945	0.08	−0.189	1

**Table 3:** Interclass correlation coefficients of different domains

Domains	Values
Self-Perception	0.120
Recreation	0.087
Social Capital	0.134
Infrastructure	0.155
Human Development	0.080
Demography	0.122
Health	0.072
Economic	0.088
Work Participation	0.060

The ICC is the proportion of variance in the observed domain that is due to differences across villages. For example, the ICC value for social capital is 0.134 which indicates the variation for this domain is due to the differences across villages. This ICC value justifies the grouping of households within the villages, showing that 13% of the total individual differences in social capital occurred at the village level and is due to the composition of villages [51]. Appreciable variation in ICC values among the domainss hows that village-level sources of variation do not operate uniformly across domains. These differences in household and village levels variations also exhibit possible differences in the relationship between these domains at two levels of analysis. These results justify the need for MFLA to extract the prevalent factor structure for this nested dataset. MFLA with varimax rotation is made on within and between correlation matrices separately to obtain factors and their respective scores at the household and the village levels [52].

The mean and standard deviation values of nine domains at household and village levels are is given in Table 4. The average values of nine domains do not vary much between levels whereas variations at the household level are on the higher side but not so at the village level. The test statistics of Table 5 show the model fit for this data is good. The averages and standard deviations of these scores of nine domains at household and village levels are given in Table 4. These averages and standard deviations are statistically significant at the household level (Table 5).

**Table 4:** Mean and Standard Deviation of nine domains at household and Village levels

Domains	Household Level (***n*** = 1096)		Village Level (***n*** = 19)	
	Mean	SD	Mean	SD
Self-Perception	0.376	0.390	0.367	0.088
Recreation	0.106	0.123	0.115	0.046
Social Capital	0.138	0.213	0.123	0.055
Infrastructure	0.450	0.170	0.460	0.077
Human Development	0.289	0.205	0.280	0.067
Demography	0.494	0.151	0.482	0.071
Health	0.437	0.246	0.446	0.040
Economic	0.221	0.143	0.225	0.025
Work Participation	0.476	0.140	0.464	0.047

**Table 5:** Model Fit characteristics of the multi-level model of quality of life

		Estimate	S.E.	Est./S.E.	***P***-Value (Two-Tailed)
**Within Level**	**Variances of Nine Domains**				
	Self-Perception	0.149	0.004	38.252	0.000
	Recreation	0.014	0.003	5.652	0.000
	Social Capital	0.045	0.003	13.335	0.000
	Infrastructure	0.025	0.002	12.236	0.000
	Human Development	0.039	0.002	17.781	0.000
	Demography	0.022	0.002	13.180	0.000
	Health	0.060	0.001	53.525	0.000
	Economic	0.020	0.001	14.453	0.000
	Work Participation	0.019	0.002	8.699	0.000
**Between Level**	**Means of Nine Domains**				
	Self-Perception	0.373	0.017	21.395	0.000
	Recreation	0.113	0.010	11.391	0.000
	Social Capital	0.131	0.011	12.132	0.000
	Infrastructure	0.457	0.017	27.579	0.000
	Human Development	0.280	0.015	18.731	0.000
	Demography	0.486	0.016	30.105	0.000
	Health	0.440	0.011	41.362	0.000
	Economic	0.222	0.006	36.598	0.000
	Work Participation	0.472	0.013	37.243	0.000
	**Variances of nine Domains**				
	Self-Perception	0.003	0.003	1.008	0.314
	Recreation	0.001	0.001	2.541	0.011
	Social Capital	0.001	0.001	1.180	0.238
	Infrastructure	0.004	0.002	2.399	0.016
	Human Development	0.003	0.001	3.104	0.002
	Demography	0.003	0.003	0.803	0.422
	Health	0.000	0.000	0.773	0.440
	Economic	0.000	0.000	2.076	0.038
	Work Participation	0.000	0.001	0.304	0.761
**Chi-Square Test of Model Fit**	Value			2299.101	
	D.F			72.000	
	*P*-Value			0.000	
	Scaling Correction Factor for MLR			0.615	
	**Standardized Root Mean Square Residual**				
	Value for Within			0.184	
	Value for Between			0.443	

### Factor analysis at the household level

The total correlation coefficient is partitioned into a household (within) and village (between) components. It is known that the sample correlation coefficient is a consistent estimator to the population within-correlation matrix and hence this within correlation matrix is submitted for exploratory, principal axis, factor analysis using M-plus software. The total variance (eigenvalues) explained by factors are given in Table 6.

**Table 6:** Total variance explained (Eigenvalues) in the multi-factor model

Components	Eigen Values	
	Within	Between
**1**	**2.403**	**4.281**
**2**	**1.474**	**2.555**
**3**	**1.168**	**1.083**
4	0.938	0.710
5	0.747	0.339
6	0.649	0.028
7	0.604	0.002
8	0.550	0.001
9	0.467	0.001

**Fig. 1:**
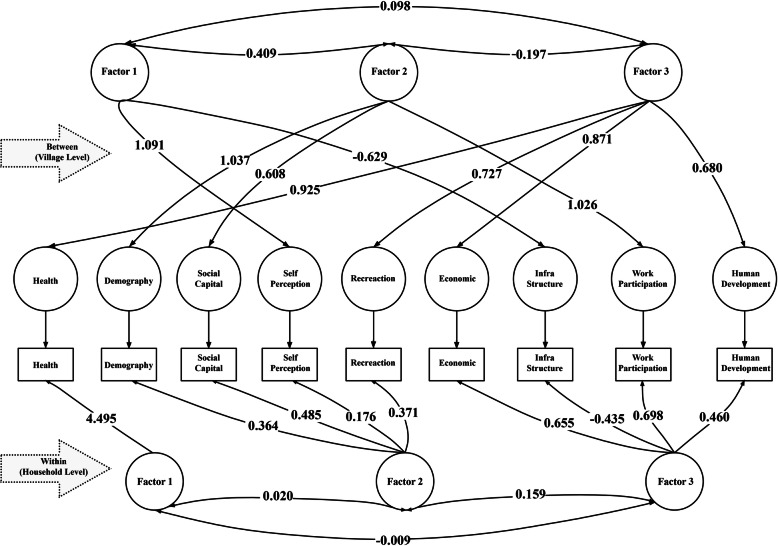
Multi-level exploratory factor analysis of the multi-factor quality of life index

The first three eigenvalues are above unity and they are 2.403, 1.474, and 1.168, and the factor loadings of these three factors are given in Table 6 and visualized in Fig. 1. All nine domains are highly loaded in the first three factors. These three factors are positively correlated among themselves (Fig. 1). The Health domain is highly loaded in the first factor, Demography, Social capital, Self-Perceived quality of life, and Recreation are accommodated in the second factor and domains like Economic Condition, Work Participation, Human Development, and Infrastructure Development are loaded highly in the third factor (Table 7).

**Table 7:** Factor loadings of the various domains at the household and village level by multilevel factor analysis

	Within (Household Level)			Between (Village Level)		
Index		Component			Component	
	**Factor 1**	**Factor 2**	**Factor 3**	**Factor 1**	**Factor 2**	**Factor 3**
Self-Perception	0.089	**0.176**	−0.044	**1.091**	−0.002	0.100
Recreation	−0.004	**0.371**	0.022	−0.030	−0.252	**0.727**
Social Capital	0.004	**0.485**	−0.042	0.456	**0.608**	−0.167
Infra. Development	−0.002	− 0.357	**− 0.435**	**− 0.629**	−0.440	− 0.003
Human Development	− 0.015	0.330	**0.460**	0.067	0.048	**0.680**
Demographic	0.010	**0.364**	0.295	−0.144	**1.037**	0.006
Health	**4.495**	0.000	0.000	0.294	0.000	**0.925**
Economic	0.018	−0.020	**0.655**	0.005	−0.330	**0.871**
Work Participation	0.001	0.002	**0.698**	0.001	**1.026**	0.202

### Factor analysis at village level

The between village correlation matrix is not a consistent estimator of the between a matrix in the population and some adjustments have to be made to extract factors from them. Factor analysis of between (Village) correlation matrixes yields three factors. The eigenvalues of these three factors are 4.281, 2.555, and 1.083 as shown in Table 6. The first factor contains high positive loading for the Self-Perceived Quality of Life domain and a stronger negative loading for the Infrastructure Development domain. The second factor is marked by three strong positive loadings on Demography, Work Participation, and Social Capital. Domains like Recreation, Health, Economic, and Development are all positively and highly loaded in the third factor (Table 7).

### Assessment of quality of life

The scores of this multilevel multifactor index can be used to assess the quality of life of people at different levels under the assumption that the higher the score better the quality of life. The quality of life of tribal people at the household and village levels is assessed separately based on the scores of this multilevel multifactor index. Then these scores are normalized as described earlier. These normalized scores generally range from zero to unity and they are also expressed in percentages. To put these scores in the frequency distribution form, the five quintile values of the score are made and they are classified as follows- less than 20% is denoted as poor, 20 to 40% as low, 40 to 60% as moderate, 60 to 80% as good and more than 80% as excellent.

The scores of the 1096 households indicate that the quality of life in 327(29.8%) households is poor, in 278 (28.4%) households is low, in 210 (19.2%) households is moderate, in 232(21.8%) households as good and in 48(4.4%) households it is excellent. This is shown in Table 8.

**Table 8:** The household-level quality of life in the surveyed villages

QoL	Frequency	Percentage
Poor	327	29.8
Low	278	25.4
Moderate	210	19.2
Good	233	21.3
Excellent	48	4.4
Total	1096	100.0

At the village level, the quality of life in two villages is marked as low, in four villages as poor, in seven villages as average, in three villages as good and finally in three villages as excellent. This result is shown in Table 9.

**Table 9:** The village level quality of life in the surveyed villages

QoL	Frequency	Percentage
Poor	2	10.5
Low	4	21.1
Moderate	7	36.8
Good	3	15.8
Excellent	3	15.8
**Total**	19	100.0

## Discussion

Quality of life is the expectation of an individual or society for leading a satisfying life and these expectations are guided by goals, values, and socio-economic factors. Indicators of quality of life include not only wealth and health but also include family, education, environment, infrastructure, and self-perception, etc. (Wikipedia). In this study, an attempt is made to develop a multi-factor index to assess the quality of life of the tribal population using multilevel factor analysis. The main advantages of using this type of analysis are that its outcome is multivariate in nature and at the same time it takes care of the hierarchical structure of the data. It provides efficient methods for estimating the stable parameters, provides the least standard errors, and does not violate the assumptions, and such facilities are not available in a separate factor analysis approach [53, 54].

In India, the tribal population is diverse groups and they are divided based on race, language, and geographical locations. Studying the wide existing disparities in these groups and improving the quality of life of these communities are some of the very important objectives of public health policy. An appropriate methodology is required to assess the quality of life of such groups. Given the multi-dimensional nature of quality of life, a multi-factor index is needed which can assess each domain of quality of life and provide suitable weight and aggregate these domains in a scientific manner [55]. This study addresses the need for such a multi-factor index to measure and analyze the quality of life of the Malayali tribal population of India. The empirical analysis of this study will provide useful information that will help us to understand the present status of this community and also help the concerned official to implement appropriate corrective measures to improve the quality of life of these communities and create an equitable society.

Significant statistics like averages and standard deviations of nine domains at the household level indicate that they play notable roles in assessing the quality of life of this tribal population. The significant variances indicate that the scores of all nine domains vary significantly from household to household. Similarly, at the village level, all average scores of nine domains are significantly different from zero but some variances are not significantly different from zero. The variances of scores of Self-Perception, Social Capital, and Demography, and Work Participation are statistically insignificant which indicates that these four domains do not vary much from village to village.

At the household, factor analysis accommodates all nine domains in the first three factors. The Health domain is highly loaded with maximum weight in the first factor and hence this factor is referred to as the Health factor. The Health domain is a composite index of Morbidity and Maternal and Reproductive Health indicators. High positive factor scores of these three domains show that better health means lesser morbidity and also good coverage of antenatal and postnatal care. The average normalized value of this index is about 0.5. That is, 50 % of household members of this study keep good health.

Substantively, the second factor appears to be a Socio-Demographic factor with very high positive loadings for four domains, namely, Demography, Social capital, Self-Perceived Quality of Life, and Recreation. Positive loadings of these four domains indicate that these four domains behave alike. This factor refers to the complex interaction of family size, family type, the family social relationships, the household’s level of recreation and leisure, and its overall perceived quality of life. This finding implies that in this tribal area, the family type and family size seem to be strongly related to the social connectedness of the family and self-perceived quality of life.

Domains like Economic Condition, Work Participation, Human Development, and Infrastructure Development are loaded highly in the third factor. The first three domains are positively loaded whereas the last one is negatively loaded in this factor and this factor is called a contrast factor. Three out four domains highly loaded in this factor are economy-related domains and hence this factor may be called an economic factor. A good Economic condition means less dependency ratio, good education, and improved employment opportunities with high income. Higher Work participation means most of the members of these households are employed. The Human Development domain consists of Wealth Status and Literacy Level. The combination of these three domains, in general, indicates that in a household if most of the members are well educated and are employed with good income then the wealth status of that household increases. But surprisingly infrastructure domain is negatively related to this combination of domains which establishes that this tribal population tends to work hard to improve their economic status even if the surrounding infrastructure is in poor condition.

At the village level, the first factor is a contrast factor that accommodates domains like Infrastructure and Quality of Life, This result shows that even when the infrastructure facility decreases, the perceived quality of life of this tribal population increases. This implies that these people as a group lead a content life with whatever infrastructure facility available for them at their villages. The second factor is marked by three strong positive loadings on Demography, Work Participation, and Social Capital. These positive loadings imply that at the village level, the jointly increasing values of the Demographic and Work Participation domains see an increase in the social capital domain also. This joint positive association between these domains shows that most of these people take employment regularly and also they become members of some social groups and intern participate in the deliberation of these groups. Recreation, Health, Economic, and Development domains are all positively and highly loaded in the third factor. That is, at the village level the increase in the education, occupation and income status makes these people lead a healthy life and they also find the time and to spend money on recreation and thereby there will be overall development in the community.

From the cross-classification Tables 10 and 11 we see that within each category of villages, the quality of life of different households varies from poor to excellent. The quality of life of 327 households is marked as a poor category because the average values of the following domains, Self-Perception, Recreation, Infrastructure, Health, and Economy are the least when compared to the average values of these domains of the households of other categories. This indicates that since the economic condition of these households is very poor, the members of these households do not have enough financial facility to contribute to the infrastructure of their villages, they are not able to maintain their health, and also do not have enough money to spend on recreation. The self-perception of the members of these households is the lowest because of these deficiencies. The average scores of the domains like Demography, Work Participation, and Human Development are the maximum for 278 households which are classified as low. It seems that the households of this category have more members in their houses and most of them are earning members and hence their Human Development Index is on the higher end. These positive aspects have elevated this category of these households from poor to low. It can be seen that the members of 210 households which are marked as moderate work hard and earn more money and spend most of the earned money on recreation. The average score of the social capital domain of 233 households classified as good is the maximum. This result shows that most of the members of the households of this category become members of the societies functioning in their villages and also participate actively in the deliberations of these societies. It seems that the economic condition and infrastructure status of 48 households which are marked as excellent are very good and the health condition and self-perception of the members of these households are very impressive. The family size of this group of households is the smallest so also the human development and the members confine themselves with their household activities only.

**Table 10:** This table provides a comparison of the quality of life at the village and household levels

			Village Level Quality of Life					Total
			Poor	Low	Moderate	Good	Excellent	
**Household Level** **Quality of Life**	Poor	Count	29	105	116	34	43	327
		%	8.9	32.1	35.5	10.4	13.1	100.0
	Low	Count	20	82	95	25	56	278
		%	7.2	29.5	34.2	9.0	20.1	100.0
	Moderate	Count	10	49	86	24	41	210
		%	4.8	23.3	41.0	11.4	19.5	100.0
	Good	Count	15	62	93	25	38	233
		%	6.5	26.6	39.9	10.7	16.3	100.0
	Excellent	Count	2	10	18	5	13	48
		%	4.2	20.8	37.5	10.4	27.1	100.0
Total		Count	76	308	408	113	191	1096
		%	7.0	28.1	37.2	10.3	17.4	100.0

**Table 11:** Household-level quality of life in the various domains

Quality of Life	Poor (***N*** = 327)	Low (***N*** = 278)	Moderate (***N*** = 210)	Good (***N*** = 233)
	μ ± S.E (μ)	μ ± S.E (μ)	μ ± S.E (μ)	μ ± S.E (μ)
Self-Perception	0.078 ± 0.012	0.374 ± 0.024	0.413 ± 0.025	**0.674 ± 0.019**
Recreation	0.093 ± 0.005	0.114 ± 0.008	**0.115 ± 0.008**	0.109 ± 0.009
Social Capital	0.108 ± 0.010	0.148 ± 0.014	0.155 ± 0.016	**0.167 ± 0.015**
Infrastructure	0.441 ± 0.009	0.449 ± 0.010	0.441 ± 0.013	**0.464 ± 0.010**
Human Development	0.273 ± 0.011	**0.318 ± 0.013**	0.292 ± 0.014	0.282 ± 0.013
Demography	0.456 ± 0.006	**0.528 ± 0.009**	0.494 ± 0.012	0.514 ± 0.010
Health	0.202 ± 0.001	0.280 ± 0.005	0.584 ± 0.008	**0.728 ± 0.006**
Economic	0.203 ± 0.007	**0.233 ± 0.009**	0.227 ± 0.010	0.215 ± 0.009
Work Participation	0.478 ± 0.007	**0.489 ± 0.008**	0.464 ± 0.011	0.470 ± 0.009

Lack of infrastructure and recreation facilities makes two villages a poor category. Four villages with good recreation facilities but very poor health status are classified as low. Good health conditions make seven villages to be classified as moderate. Three villages with poor economic conditions and the Human Development Index are de-promoted from excellent to good status. Impressive economic and infrastructure conditions and a good Human Development Index make three villages to be marked as excellent as shown in Tables 10, 11, and 12.

**Table 12:** Village-level quality of life in the various domains

Quality of Life	Poor (***N*** = 2)	Low (***N*** = 4)	Moderate (***N*** = 7)	Good (***N*** = 3)
	μ ± S.E (μ)	μ ± S.E (μ)	μ ± S.E (μ)	μ ± S.E (μ)
Self-Perception	0.292 ± 0.043	0.400 ± 0.023	**0.404 ± 0.019**	0.372 ± 0.037
Recreation	0.066 ± 0.009	**0.115 ± 0.007**	0.104 ± 0.005	0.104 ± 0.011
Social Capital	0.120 ± 0.022	**0.153 ± 0.013**	0.147 ± 0.011	0.141 ± 0.018
Infrastructure	0.401 ± 0.017	0.410 ± 0.008	0.438 ± 0.008	**0.498 ± 0.015**
Human Development	0.263 ± 0.023	**0.303 ± 0.011**	0.277 ± 0.01	0.233 ± 0.018
Demography	**0.504 ± 0.014**	0.498 ± 0.008	0.494 ± 0.008	0.501 ± 0.015
Health	0.425 ± 0.028	0.411 ± 0.014	**0.453 ± 0.012**	0.440 ± 0.023
Economic	**0.226 ± 0.015**	0.218 ± 0.008	0.214 ± 0.007	0.198 ± 0.011
Work Participation	0.475 ± 0.015	**0.485 ± 0.007**	0.478 ± 0.007	0.459 ± 0.015

A similar study was undertaken by Milind Kumar Yadav and Ajeet Kumar on the Tharu tribe resides in Lakhimpur Kheri district of Uttar Pradesh, India identifies that occupation, land ownership, and family size are the key factors responsible for determining the household income and the quality of life this tribal population [56]. The adolescents of tribes of Jawadhi hills of Tamil Nadu, India preferred to have good physical, psychological, and environmental well beings and meaningful social relationships to lead a satisfying quality of life [57]. According to Sitakant Mahapatra, in general, the Indian tribal belief of good and happy life is integrally linked to his/her view of culture which emphasizes health and disease-free life, love of fun, a reasonable degree of freedom and leisure, an intimate balance between individual existence and the natural, social, and the supernatural orders [58]. Lack of health care Centers, lack of health sensitization, low literacy, poverty, and malnourishment, etc. are some of the factors worsening the quality of life of the tribal population of Western Ghats of India [59].

This type of multi-factor index developed based on realistic data will help planners, developers, and policymakers to monitor the key factors that encompass the social, health, environmental, and economic dimensions of quality of life at the household and community levels of these tribal people.

## Conclusion

The main aim of this study was to assess the quality of life of the Malayali tribal population of Tamil Nadu State at the household and at the village, levels using nine domains, namely, Demography, Economy, Health, Human Development, Infrastructure development, Work Participation, Recreation, Social Capital and Self Perception. To accomplish this task, multilevel factor analysis was performed to extract uncorrelated meaningful factors and their factor scores from the nested structure dataset using Mplus software. A multi-level multifactor index was constructed using these factors and their respective weights. The quality of life of different households and different villages were assessed using the scores of this index. Thus this study proposes a step-by-step procedure to develop an index and to assess the quality of life of individuals and communities based on this index. The main purpose of developing such an index is to provide a tool for tribal development based on realistic data that can be used to monitor the key factors that encompass the social, health, environmental, and economic dimensions of quality of lives at the individual, household and community levels of these tribal people.

## Supplementary Information

**Additional file 1.**

## Data Availability

The details of variables, descriptive and multilevel analyses are available from the author on reasonable request. We developed and tested the questionnaire for this study and it is attached herewith as a supplementary copy.
