# Supplementary material for: Development of multifactor index for assessing quality of life of a tribal population of India: multilevel analysis approach
Source: BMC Public Health. 2021 Feb 18;21:383. doi: 10.1186/s12889-021-10338-2 (PMC7893862; doi:10.1186/s12889-021-10338-2)
Supplement: Supplementary file 1 — Additional file 1. [file 12889_2021_10338_MOESM1_ESM.pdf]

(Confidential for Research purpose only)

School of Public Health, SRM University
Quality of life of Tribal People:
Development of Individual, Household and Community based Multi-Factor Indices
Funded By ICMR
பழங்குடி மக்களின் வாழ்க்கைத் தரம்: தனிநபர்,குடும்பம் மற்றும் சமூகம் சார்ந்த பன்முக குறியீடுகள் உருவாக்கம்

I1. Household Questionnaire Number: ☐☐☐☐
(குடும்ப வினாத்தாள் வரிசை எண்)

I2. State<br>மாநிலம்	தமிழ்நாடு	I3. District:<br>மாவட்டம்	
I4. Taluk<br>வட்டாரம்		I5. Town/Village/நகரம்/கிராமம<br>பெயர்	
I6. Door No<br>கதவு எண்		I7. Name of Household Head/குடும்ப தலைவர் பெயர்	
I8. Tribe Name /<br>மலைவாழ்<br>மக்கள் பெயர்		I9. Sub Tribe Name/மலைவாழ்<br>மக்கள் துணைப்பெயர்	

I10. Status of interview (பேட்டியின் முடிவு(

	No of Eligible Respondents<br>தகுதி வாய்ந்த பதிலளிப்பவர் எண்ணிக்கை	Completed<br>பூர்த்தி செய்யப்பட்டவை	No Eligible respondent<br>தகுதி வாய்ந்த பதிலளிப்பவர் இல்லை	Refused<br>மறுப்பு	Others<br>மற்றவை
Household (Head)<br>குடும்பத் தலைவர்					
Women (Age 15 - 49)<br>பெண்கள்) 15 வயது 49 முதல் வயது வரை(					
Male (> 19) ஆண்கள் (19 வயதிற்க்கு மேற்பட்டோர்(					
Adolescent(≤19)<br>விடலைப் பருவம்) 19 வயதிற்கு கீழ் உள்ளோர்(					
Elders (≥ 60)<br>முதியோர் (60 வயதிற்கு மேற்பட்டோர்(					

I11. Line No of Respondent to Household Questionnaire<br>(குடும்ப வினாத்தாளுக்கு பதிலளிப்பவரின் வரிசை எண்	☐☐
I12. Total Number of Household Members<br>வீட்டு உறுப்பினர் எண்ணிக்கை	☐☐

I13. Date of Interview<br>பேட்டி எடுக்கும் தேதி		I14. Time of Interview<br>பேட்டி எடுக்கும் நேரம்	
I15. Field Investigator Code<br>கள ஆய்வாளரின் குறியீடு		I16. Supervisor Code<br>கண்காணிப்பாளரின் குறியீடு	

Household Member Details (குடும்ப உறுப்பினர்கள் விபரம்)

Line no வ.எண்	H1	H2	H3	H4	H5	H6	H7	H8	H9	H10	H11	H12	H13	H14	H15
	Name பெயர்	Gender பாலினம்	Marital Status திருமண விபரம்	Age வயது	Can Read and write ? எழுத படிக்க தெரியுமா?	[0–Skip to H12]<br>Educational Status கல்வித் தகுதி	[Yes – Skip to H10]<br>Is the person pursuing education ? தற்சமயம் படித்து கொண்டு-ள்ளாரா?	Year of completion முடித்த வருடம்	Reason for Discontinuation ? கல்வி தொடராமைக்கு காரணம்?	Educational Institution category? கல்வி நிறுவன வகை?	Distance to education facility? கல்வி நிறுவனத்தின் தொலைவு?	Main occupation ? முதன்மை தொழில்?	Monthly income from all sources ? ஒட்டு மொத்த மாத வருமானம்	Height உயரம்	Weight எடை

Code for (H2)

01=Male ஆண்
02=Female பெண்

Code for (H3)

01=Single மணமாகாதவர்
02=Married மணமானவர்
03=Other /மற்றவை

Code for (H5)

01=Yes ஆம்
02=No இல்லை

Code for (H7)

01=Yes ஆம்
02=No இல்லை

Code for (H9)

01=Education not considered as necessary/கல்வி அவசியம் என கருதவில்லை
02=Required for household work / family business/care of siblings/குடும்ப வேலையினால்/ குலத்தொழில் செய்ய / சகோதரர்களை கவனித்துக் கொள்ள
03=Need to work for payment or kind/பணம் அல்லது உணவுக்காக வேலை செய்ய வேண்டி நேர்ந்தது
04= Repeated failures/அடுத்தடுத்த தோல்விகள்
05= Got married/ திருமணம்
06= Others/ மற்றவைகள்

Code for (H10)

01= Government/அரசாங்கம்
02= Private/தனியார்
03= Anganwadi/பால்வாடி
04=Tribal/social welfare schools/மலைவாழ் மக்கள் / சமூக நலப் பள்ளி
05= NGO schools (open schools)/அரசு சாரா நிறுவன பள்ளி

Code for (H11)

01= Foot/நடந்து
02= Cycle/மிதிவண்டி
03= Motorized Transport மோட்டர்/வாகனம்
04= Public Transportation/
05 = other/மற்றவை

Code for (HH12)

01= Agriculture/விவசாயம்
02=Labour /கூலி வேலை
03=Salaried Employment/ஊதியம் சார்ந்த வேலைவாய்ப்பு
04=Business/ வியாபாரம்
05=Unemployed/வேலையில்லாதவர்
06=Student/மாணவர்
07=House wife/இல்லத்தரசி
08=Retired/ஓய்வுபெற்றவர்
09=Others/மற்றவைகள்

HOUSEHOLD CHARACTERISTICS

Q.NO	QUESTIONS	CATEGORIES	CODING	SKIP TO
H16	Type of family (குடும்ப வகை)	Nuclear Family/தனிக் குடும்பம் ______ 1<br>Joint Family/கூட்டுக் குடும்பம் ______ 2		
H17	Religion<br>மதம்	Hindu/ இந்து ______ 1<br>Muslim/முஸ்லிம் ______ 2<br>Christian/கிறிஸ்டியன் ______ 3<br>Others/மற்றவர்கள் ______ 4		
H18	Do you or any member of your household own this house? உங்களுக்கோ அல்லது குடும்பத்தில் உள்ள மற்றவருக்கோ இது சொந்த வீடா?	Own house/ சொந்த வீடு ______ 1<br>Tenant வாடகை ______ 2<br>Others மற்றவை ______ 3<br>(குறிப்பிடவும்) (mention)<br>…………………………………………………………………………...		
H19	How many rooms are there?<br>வீட்டில் எத்தனை அறைகள் உள்ளன?	Number of rooms<br>அறைகளின் எண்ணிக்கை	[ ][ ]	
H20	Type of House (Record Observation)<br>வீட்டின் அமைப்பு (பார்த்து பதிவு செய்க)	Pucca /பக்கா ______ 1<br>Semi Pucca /செமி பக்கா ______ 2<br>Kachcha/கச்சா ______ 3		
H21	What fuel is used for cooking?<br>சமைப்பதற்கு என்ன வகையான எரிபொருள் பயன்படுத்தப்படுகிறது?	LP Gas/சமையல் எரிவாயு ______ 1<br>Kerosene/மண்ணெண்ணெய் ______ 2<br>Wood or charcoal/ விறகு / கரி ______ 3<br>Dung Cake /வரட்டி ______ 4<br>Other(specify / மற்றவை/குறிப்பிடுக) ______ 5<br>…………………………………………………………………………...		
H22a	Does your household have electricity? உங்கள் வீட்டில் மின்சாரவசதி உள்ளதா?	Yes/ஆம் ______ 1 →<br>No/இல்லை ______ 2		H23
H22b	Source of lighting<br>வெளிச்சதிற்கான மூலதனம்	Gas/எரிவாயு ______ 1<br>Oil//எண்ணெய் ______ 2<br>Kerosene/மண்ணெண்ணெய் ______ 3<br>Others(மற்றவை) ______ 4<br>…………………………………………………………………………		
H23	Is there a kitchen in your house? உங்கள் வீட்டில் சமையலறை உள்ளதா?	Yes/ஆம் ______ 1<br>No/இல்லை ______ 2		
H24	Where do you usually cook?<br>வழக்கமாக சமையல் எங்கு செய்யப்படுகிறது?	In the house/வீட்டில் ______ 1<br>In a separate building/வேறு வீட்டில் ______ 2<br>Outdoors/வெளியே ______ 3<br>Other/மற்றவை ______ 4<br>(குறிப்பிடுக)………………………………………………………………		

			Yes	No	Count
H25	List the things you have in your household:<br>உங்கள் வீட்டில் உள்ள பொருள்கள்	Radio / வானொலி	1	2	
		Television (colour)/ வண்ண தொலைக்காட்சி	1	2	
		Landline/ தொலைபேசி	1	2	
		Mobile telephone /கைப்பேசி	1	2	
		Ceiling fan /மின்விசிறி	1	2	
		Table fan /மேசை விசிறி	1	2	
		Chair /நாற்காலி	1	2	
		Cot/Bed கட்டில் / படுக்கை	1	2	
		Mattress /மெத்தை	1	2	
		Sewing machine/ தையல் இயந்திரம்	1	2	
		Pressure cooker/ குக்கர்	1	2	
		Grinder /அரைக்கும் இயந்திரம்	1	2	
		Mixie /மிக்சி	1	2	
		DVD/VCD Player /டிவிடி ப்ளேயர்	1	2	
		DTH/டிஷ் ஆண்டெனா	1	2	
		Table/மேசை	1	2	
		Wall clock/ சுவர் கடிகாரம்	1	2	
		Wardrobe/ பீரோ	1	2	
		Bicycle/ மிதிவண்டி	1	2	
		Water pump தண்ணீர்	1	2	
		Motorcycle or Scooter இருசக்கர வாகனம்	1	2	
		Animal-drawn Cart விலங்கு இழுக்கும் வண்டி	1	2	
		Tractor / ட்ராக்டர்	1	2	
		Car/Jeep/JCB/Truck / கார் / ஜீப்	1	2	
		Other/மற்றவை(குறுப்பிடவும்)<br>…………………………………………………………………………			

H26	Does your household own any farming lands?<br>இந்த குடும்பத்திற்கு ஏதேனும் விவசாய நிலம் உள்ளதா?	Yes/ ஆம்____________ 1<br>No/ இல்லை____________2 →	H29
H27	How much area of farming land does this household own?<br>எவ்வளவு விவசாய நிலம் உள்ளது?	Acres (ஏக்கர்)<br>If not in acres; specify in some Unit<br>(ஏக்கரில் இல்லையெனில் மற்ற அலகில் குறிப்பிடவும்)	☐☐☐ ________	
H28	How much area of this farm is irrigated?<br>இதில் எவ்வளவு நீர் பாயக்ககூடியது?	Acres(ஏக்கர்)<br>If not in acres; specify in some Unit<br>(ஏக்கரில் இல்லையெனில் மற்ற அலகில் குறிப்பிடவும்)	☐☐☐ ________	
H29	Does your household own any livestock?<br>இந்த வீட்டில் ஏதேனும் ஆடு, மாடு, கோழி போன்ற விலங்குகள் உள்ளனவா?	Yes/ஆம் ____________1<br>No/இல்லை____________2 →	H31

H30	What are all the animals in the list?<br>பட்டியலில் உள்ள என்னென்ன விலங்குகள் உள்ளன?	(see sub-table below)	
H31	Does anyone in this household hold a savings account / bank savings account / postal savings account? குடும்பத்தில் யாரேனும் வங்கி, தபால் அலுவலக அல்லது சிறுசேமிப்பு கணக்கு வைத்துள்ளார்களா?	Yes/ஆம்____1<br>No/இல்லை____2	→ H33
H32	How many members have the account? எத்தனை பேர் வைத்துள்ளார்கள்?	Male /ஆண்கள் [ ][ ]<br>Female/பெண்கள் [ ][ ]	
H33	Does anyone in your household read newspaper? குடும்பதில் யாருக்கேனும் செய்தித்தாள் படிக்கும் வழக்கம் உள்ளதா?	Yes/ஆம்____1<br>No/இல்லை____2	
H34	What is the main source of water supply for this family? குடும்பத்திற்கான முக்கிய நீர் ஆதாரம் என்ன?	Piped water in residence குழாய் தண்ணீர் (வீட்டிலேயே)____________1<br>Well water in residence கிணற்று தண்ணீர்(வீட்டிலேயே)___________ 2<br>Public tap in residence/பொது குழாய் (வீட்டிலேயே) _______________3<br>Public tap/ பொது குழாய் ____________________________________ 4<br>Public well water / பொது கிணறு____________________________ _5<br>Pond/குளம்____6<br>Tanker truck தண்ணி லாரி_________________________________7<br>Others/மற்றவை____ 8<br>(குறுப்பிடுக)	
H35	Does your household get uninterrupted water supply? உங்கள் வீட்டில் தங்குதடையின்றி தண்ணீர் வரத்து உள்ளதா?	Yes/ஆம்____1<br>No/இல்லை____2	
H36	How much time does it take to collect/fetch water? தண்ணீர் சேகரிக்க/ பிடிக்க எவ்வளவு நேரம் ஆகும்?		
H37	From your family, who regularly fetches water? உங்கள் குடும்பத்தில் வழக்கமாக யார் தண்ணீர் பிடிக்க செல்வார்கள்?	Woman/பெண்கள் ____ 1<br>Man /ஆண்கள்____ 2<br>Children /குழந்தைகள்___________________________________ 3<br>All/அனைவரும் ____4	

H30 sub-table:

	Yes	No
Cows/Buffalos/Bulls-மாடு/எருமை/காளை	1	2
Horses/Donkeys குதிரை/கழுதை	1	2
Goats/வெள்ளாடு	1	2
Sheep/செம்மறியாடு	1	2
Chickens/ Ducks /கோழி/வாத்து	1	2
Pigs/பன்றி	1	2

H38	Do you treat your water to make it safer to drink?<br>குடிப்பதற்கு பாதுகாப்பான தண்ணீரை பெறும் வகையில் உங்கள் தண்ணீரை எதாவது வழிமுறையில் சுத்திகரிப்பு செய்கிறீர்களா?	Yes/ஆம் ______ 1<br>No/இல்லை______ 2 →<br>Occasional/ எப்போதாவது ______3	H40
H39	How do you treat your drinking water?<br>குடிநீரை சுத்தபடுத்த என்ன செய்வீர்கள்?	Strain by cloth துணியால் வடிகட்டுதல்______1<br>Use a water filter தண்ணீர் வடிகட்டி பயன்படுத்துதல்______ 2<br>Boiling கொதிக்கவைத்தல்______3<br>Others/மற்றவை______4<br>(குறிப்பிடுக)..	
H40	Does your house have toilet facilities?உங்கள் வீட்டில் கழிப்பிட வசதி உள்ளதா?	Yes/ஆம்______1<br>No /இல்லை______2 →	H43
H41	What type of toilet facility is there?<br>என்ன வகையான கழிப்பிட வசதி உள்ளது?	Flush Latrine / ப்ளஷ் டாய்லெட்______ 1<br>Septic Tank / செப்டிக் டேங்க் ______ 2<br>Pit latrine / குழி டாய்லெட் ______ 3<br>Others specify/மற்றவை ______4<br>(குறிப்பிடுக)..	
H42	How many household members use this toilet facility?<br>வீட்டில் உள்ள எத்தனை பேர் கழிப்பிட வசதியை பயன்படுதுகிறார்கள்?	All / அனைவரும்______1<br>Men/ஆண்கள்______2<br>Women/பெண்கள்______ 3<br>Children /குழந்தைகள்______4	
H43	Where do you go for defecation/நீங்கள் எங்கே மலம் கழிபீர்கள்?	Open/திறந்தவெளி______1<br>Public Toilet /பொது கழிப்பிடம்______2<br>Others specify/மற்றவை______3<br>(குறிப்பிடுக)	
H44	Does your house have drainage facility?<br>உங்கள் வீட்டில் சாக்கடை வசதி உள்ளதா?	Yes/ஆம் ______ 1<br>No/இல்லை______ 2→	H46
H45	Type of the drainage?<br>சாக்கடை வசதியின் வகை	Closed/மூடிய வகை______1<br>Open / திறந்த வகை______2<br>Others / மற்றவை______3	
H46	How do you remove/dispose the household waste?<br>உங்கள் வீட்டு குப்பைகளை எவ்வாறு அப்புறப்படுதுவீர்கள்?	Public container/ பொது குப்பை தொட்டி______1<br>Open Dump/ திறந்த வெளியில் கொட்டுதல்______2<br>Other ways/மற்றவை______3<br>(குறுப்பிடவும்)	
H47	Did any member of this household marry in the past year 1ST jan to 31 ST Dec 2011? கடந்த ஒரு வருடத்தில் இந்த குடும்பத்தை சார்ந்த எவருக்கேனும் திருமணம் நடந்ததா?	Yes /ஆம்______1<br>No/இல்லை______2 →	H50
H48	How many marriages were there from 1ST jan to 31 ST Dec 2011? எத்தனை திருமணங்கள் நடந்தன?	Boys/ஆண்கள் ___<br>Girls/பெண்கள் ___<br>Total/மொத்தம் ___	
H49	What was his/her age at the time of marriage?<br>திருமணத்தின் போது அவர்களுடைய வயது என்ன?	Boy / Girl<br>1<br>2<br>3<br>4	

H50	Was there a birth in your family/household in the past year 1ST jan to 31 ST Dec 2011? கடந்த ஒரு வருடத்தில் உங்கள் வீட்டில் ஏதேனும் பிறப்பு நடந்ததா?	Yes/ ஆம்____________________1<br>No/இல்லை____________________2→	H52
H51	How many births?<br>எத்தனை பிறப்புகள்?	Male ஆண் | Female பெண் | Total மொத்தம்<br>___ | ___ | ___	
H52	Was there a death in your family/household in the past year? From 1ST jan to 31 ST Dec 2011; கடந்த ஒரு வருடத்தில் உங்கள் வீட்டில் ஏதேனும் ,இறப்பு நடந்ததா?	Yes/ ஆம்____________________1<br>No/இல்லை____________________2 →	H68
H53	How many deaths?<br>எத்தனை இறப்புகள்?	Male ஆண் | Female பெண் | Total மொத்தம்<br>___ | ___ | ___	

H54	H55	H56	H57	H58	H59	H60	H61	H62
What (was/were) the names of the persons who died? இறந்தவர்களின் பெயர்கள் என்ன?	Male(=1) ஆண் / Female பெண்(=2) பாலினம்	How old was she /he when died<br>1.Enter in days if died in less than 30days(பிறந்த 30 நாட்களுக்கு முன்பாகவே இறந்திருந்தால், நாட்களில் குறுபிடுக)<br>2. Enter in months if died in less than 12 months(பிறந்த 12 மாதங்களுக்கு முன்பாகவே இறந்திருந்தால், மாதகளில் குறுபிடுக)<br>3. others enter in years (மற்றவைகளை வருடத்தில் குறுபிடுக)	In what month and year did (N) die?<br>இறந்த மாதம் மற்றும் வருடம்என்ன?	Check whether deceased female aged 15-49 was at the time of death?<br>இறந்தது பெண்ணாக இருந்தால் இறந்த போது அவரின் வயது 15-49-க்குள் இருகிறதா என்பதை கவனி	If deceased was female aged 15-49 at the time of death இறந்தவர் பெண்ணாக மற்றும் இறந்த போது வயது 15-49 -க்குள் இருந்தால்			
					was (name) pregnant when She died? Probe:<br>இறக்கும் போது கர்பமாக இருந்தரா?	Did (name) die at the time of abortion or within six weeks of Abortion?<br>கருசிதைவின் போது அல்லது கரு சிதைந்த ஆறு வாரத்திற்குள் இறந்தாரா?	Did (name) die during child birth?<br>குழந்தை பிறக்கும்போது இறந்தாரா?	did (name) die within six weeks of the end of pregnancy or Child birth?<br>குழந்தை பிறந்த ஆறு வாரத்திற்குள் இறந்தாரா?
01 ________ (Name)	1<br>2	Days ☐<br>Months ☐<br>Years ☐	Month ☐<br>Year ☐	Yes ---1→<br>No----2 Go to next death	Yes ----1 Go to next Death<br>No →	Yes------1 Go to next Death<br>No →	Yes-----1 Go to next Death<br>No →	Yes------1<br>No-------2 (Go to next death)
02 ________ (Name)	1<br>2	Days ☐<br>Months ☐<br>Years ☐	Month ☐<br>Year ☐	Yes ---1<br>No ---2 Go to next death	Yes—1 Go to next Death<br>No →	Yes---1 Go to next Death<br>No →	Yes----1 Go to next death<br>No →	Yes-----1<br>No-----2 Go to next death
03 ________ (Name)	1<br>2	Days ☐<br>Months ☐<br>Years ☐	Month ☐<br>Year ☐	Yes---1 →<br>No---2 Go to next death	Yes---1 Go to next death<br>No →	Yes----1 Go to next death<br>No →	Yes---1 Go to next death<br>No →	Yes-----1<br>No-----2 Go to next death

04 ______ (Name)	1<br>2	Days [ ]<br>Months [ ]<br>Years [ ]	Month [ ]<br>Year [ ]	Yes---1 →<br>No<br>Go to next death	Yes---1<br>Go to next death<br>No →	Yes----1<br>Go to next death<br>No →	Yes-----1<br>Go to next death<br>No →	Yes-------1<br>No-------2<br>Go to next death
05__________ (Name)	1<br>2	Days [ ]<br>Months [ ]<br>Years [ ]	Month [ ]<br>Year [ ]	Yes---1 →<br>No---2<br>Go to next death	Yes----1<br>Go to next Death<br>No →	Yes----1<br>Go to next Death<br>No →	Yes-----1<br>Go to next Death<br>No →	Yes--------1<br>No---------2<br>Go to next death

Ask only in case of Maternal Death

Q. No.	Questions and filter	Coding categories	For Death 1st	For Death 2nd
	Serial No of H54<br>வரிசை எண்			
H63	What is the parity?<br>எத்தனாவது கர்ப்பம்?			
H64	How many months was she pregnant/after how many days/months of delivery did she die?<br>அவர் இறக்கும் போது எத்தனை மாத கர்ப்பிணி / பிரசவித்த பிறகு எத்தனை நாள் / மாதம் கழித்து இறந்தார்?			
H65	Where did the delivery/abortion take place? (maternal death during pregnancy details need not be collected)<br>பிரசவம் / கருச்சிதைவு எங்கு நடைபெற்றது?(கர்ப்பத்தின் போது இறந்த பெண்கள் பற்றிய விபரம்தேவையில்லை)	Sub-centre<br>துணை மையம்____________________<br>PHC ஆரம்ப சுகாதார மையம்____________________________.<br>Taluk hospital தாலுகா மருத்துவமனை___________________<br>District Government hospital மாவட்ட அரசு மருத்துவமனை_______________<br>Private hospital/NGO தனியார் மருத்துவமனை./ அரசு சாரா நிறுவனம்<br>Home / வீடு_____________________<br>Others / மற்றவை_________________<br>………………………………………..	1<br>2<br>3<br>4<br>5<br>6	
H66	How many days after delivery/abortion did she die?<br>பிரசவித்த / கருகலைப்புக்கு பிறகு எத்தனை நாட்கள் கழித்து இறந்தார்?	Died during pregnancy.<br>கர்ப்பத்தின் போது இறந்தார்__________<br>Died during delivery on the same day<br>பிரசவித்த அன்றே இறந்தார்__________<br>Days/ நாட்கள்_____________________	1<br>2	
H67	During pregnancy/ before delivery/after delivery /after abortion did she suffer from any of these illnesses?<br>கர்ப்பத்தின் போது / பிரசவத்திற்கு முன்பும் / பின்பும் / கருகலைப்புக்குபிறகும் அவருக்கு பின்வரும் சுகாதார பிரச்சனைகள் இருந்ததா?	**Pregnancy**(கர்ப்பத்தின் போது)	Yes No Dk	Yes No Dk
		Swelling of Hands And Feet கைகள் மற்றும் கால்களில் வீக்கம்___________	1 2 9	1 2 9
		Paleness வெளிரிய தன்மை _______	1 2 9	1 2 9
		Visual Disturbances பார்வை தொந்தரவு________________________	1 2 9	1 2 9
		Bleeding இரத்தப்போக்கு___________________.	1 2 9	1 2 9
		Convulsions வலிப்பு__________________________..	1 2 9	1 2 9
		Weak or no movement of foetus பலவீனம் அல்லது கரு இயக்கமின்மை____________________	1 2 9	1 2 9
		Abnormal position of foetus. கருவில் வழக்கத்திற்கு மாறான நிலை_____________________	1 2 9	1 2 9

		**Delivery**(பிரசவம்)		
		Premature Labour குறைப் பிரசவம்____	1 2 9	1 2 9
		Prolonged Labour(>12 hours) நீண்ட நேர பிரசவம்_____________________	1 2 9	1 2 9
		Breech Presentation தலை திரும்பி பிறத்தல்________________________	1 2 9	1 2 9
		**Post Delivery / Abortion**		
		High fever அதிக காய்ச்சல்___________	1 2 9	1 2 9
		Lower abdominal pain கீழ் வயிற்று வலி____________________________	1 2 9	1 2 9
		Foul smelling vaginal discharge துர்நாற்றமுடைய வெள்ளை படுதல் / சீழ்படுதல்________________________	1 2 9	1 2 9
		Excessive Bleeding அதிக ரத்தபோக்கு_________________	1 2 9	1 2 9
		Dizziness மயக்க உணர்வு___________________	1 2 9	1 2 9
		Severe Headache கடுமையான தலைவலி________________________	1 2 9	1 2 9

Out-Migration / இடம்பெயர்தல்							
	H68	H69	H70	H71	H72	H73	H74
L. No	Name of the member/s of this family who live/s outside (including women and children)(minimum one year and above)இந்த குடும்பத்தை சார்ந்த, வெளியில் வசிக்கும் நபரின் பெயர்கள்(பெண்கள் மற்றும் குழந்தைகள் உயட்பட)(குறைபட்சம் ஒரு வருடம் அல்லது அதற்கு மேல்)	Sex Male-1 Female-2 பாலினம் ஆண் - பெண்	Age வயது	Name of the district/state this person is currently residing at தற்சமயம் இந்த நபர் வசிக்கும் மாவட்டம் / மாநிலம்	Date and month on which this person left this village permanently இந்த நபர் நிரந்திரமாக கிராமத்தை விட்டு வெளியேறிய வருடம் மற்றும் மாதம்	What is the frequency of their visit? எவ்வளவு நாள்களுக்கு ஒரு முறை வீட்டிற்கு வந்து செல்வர்?	Reason for migration? இடம் பெயர்ந்ததின் காரணம்

1. Employment (வேலை வாய்ப்பு) 2. Seasonal employment (பருவ கால வேலை வாய்ப்பு 3.Education (கல்வி)

HEALTH

MORBIDITY (household)

H75	Did anyone in the household fall ill in the past 1 month?<br>கடந்த ஒரு மாதா காலத்தில் வீட்டில் யாருக்கேனும் உடல்நிலை சரியில்லாமல் இருந்ததா?	Yes /ஆம் ________________________<br>No/ இல்லை ______________________	1<br>2	

H76	H77	H78	H79	H80
Line Number from HH Questionnaire<br>வரிசை எண்	What was the illness?<br>என்ன மாதிரியான உடல்நலக்குறைவு?	Duration of illness (days)<br>நோய் காலம் (நாட்களில்)	Did he/she seek treatment?<br>அவர் / அவள் ஏதேனும் சிகிச்சை பெற்றாரா?	Where did he/she seek treatment?<br>எங்கே சிகிச்சை பெற்றார்?

Code for H79
Yes/ ஆம் – 1
No/ இல்லை – 2

Code for H80
Government Hospital/அரசு மருத்துவமனை - 1
PHC/ ஆரம்ப சுகாதார மையம் – 2
Sub center/துணை சுகாதாரமையம் - 3
VHN/கிராம சுகாதார செவிலியர் – 4
Private Doctor/தனியார் மருத்துவர் – 5
Private Hospital/தனியார் வைத்தியசாலை – 6
Traditional healer/பாரம்பரிய மருத்துவர்-7
Home remedies/ வீட்டு வைத்தியம் - 8

SATISFACTION WITH HEALTH FACILITY – H81

In the past 30 days did you visit any of the following health centers?
கடந்த 30 நாட்களில் கீழ்க்கண்ட ஏதேனும் நலமையதிற்கு சென்றீர்களா?

	Yes ஆம்	No இல்லை
Health Sub center? துணை சுகாதார மையம்	1	2
PHC? ஆரம்ப சுகாதார மையம்	1	2
Taluk Hospital தாலுகா மருத்துவமனை	1	2
Government Hospital அரசு மருத்துவமனை	1	2
Private Hospital தனியார் வைத்தியசாலை	1	2
Private Doctor தனியார் மருத்துவர்	1	2
NGO Hospital அரசு சாரா நிறுவனம்	1	2
Traditional healer நாட்டு வைத்தியர்	1	2

How will you rate the health facility that you visited last on the following criteria?
கடைசியாக சென்ற நல மையத்தை எவ்வாறு மதிப்பிடுவீர்கள்?

Q. No	Statement கூற்று	Bad மோசம்	Ok சுமார்	Good நன்று
H82	Location அமைவிட வசதி	1	2	3
H83	Waiting time காத்திருக்கும் நேரம்	Long 1	Average 2	No waiting 3
H84	Manners of the physician மருத்துவர் தங்களிடம் நடந்துகொள்ளும் விதம்	1	2	3
H85	Talent/Skill of the physician மருத்துவரின் திறமை	1	2	3
H86	Manners of the nurse நர்ஸ் தங்களிடம் நடந்துகொள்ளும் விதம்	1	2	3
H87	Talent/skill of the nurse நர்ஸின் திறமை	1	2	3
H88	Talent/skill of the supporting staff அங்கு வேலை செய்யும் மற்றவர்களின் திறமை	1	2	3
H89	Manners of the supporting staff அங்கு வேலை செய்யும் மற்றவர்களின் நடத்தை	1	2	3
H90	Explanation of the treatment உங்களுக்கு என்ன செய்தார்கள் என்பதை பற்றிய விளக்கம்	1	2	3
H91	Diagnostic and surgical instruments மருத்துவ அறுவை சிகிச்சை மற்றும் நோய் கண்டறியும் உபகரணங்கள்	1	2	3

Self perception தன் உணர்வு					
Q.No	Your perception about the following கீழ்க்கண்டவை பற்றி உங்களுடைய தன் உணர்வு என்ன?	Bad	OK	Good	Reason
H92	Health / உடல்நிலை	1	2	3	
H93	Education / கல்வி	1	2	3	
H94	Income / வருமானம்	1	2	3	
H95	Employment / வேலைவாய்ப்பு	1	2	3	
H96	Household facilities / வீட்டுவசதி	1	2	3	
H97	Water facilities / தண்ணீர் வசதி	1	2	3	
H98	Drainage facilities /சாக்கடை வசதி	1	2	3	
H99	Hygiene facility / சுகாதார வசதி	1	2	3	
H100	Smoking/புகை பிடித்தல்	1	2	3	
H101	Drinking/மது அருந்துதல்	1	2	3	
H102	Tobacco Chewing/புகையிலை	1	2	3	
H103	Peoples participation in community development/சமுக முன்னேற்றத்துக்கு மக்களின் பங்கெடுப்பு	1	2	3	
H104	Recreation time / பொழுதுபோக்கு நேரம்	1	2	3	
H105	Leisure time / ஓய்வு நேரம்	1	2	3	
H106	Who do you think has more leisure time? யாருக்கு அதிக ஒய்வு நேரம் இருப்பதாக நினைக்கிறீர்கள்?	Men ஆண்கள் ________________ 1<br>Women பெண்கள் ______________ 2<br>Children குழந்தைகள் ___________ 3<br>Elderly முதியவர்கள் ___________ 4			
